# Recent advances in spontaneous intracerebral hemorrhage

**DOI:** 10.12688/f1000research.16357.1

**Published:** 2019-03-18

**Authors:** Ravi Garg, José Biller

**Affiliations:** 1 Department of Neurology, Loyola University Chicago, Stritch School of Medicine , Maywood, IL, 60153, USA

**Keywords:** intracerebral hemorrhage, hematoma expansion, blood pressure lowering, antithrombotic reversal, deompressive hemicraniectomy, intraventricular alteplase

## Abstract

Intracerebral hemorrhage (ICH) is a stroke subtype associated with significant morbidity and mortality. The purpose of this review is to provide an update on important research on ICH over the past three years. Topics covered include risk factors, imaging predictors of hematoma expansion, scoring schema to predict hematoma expansion, hemostatic therapies, acute blood pressure lowering, intraventricular administration of alteplase for intraventricular hemorrhage, and the current status of surgical therapies.

## Introduction

Intracerebral hemorrhage (ICH) accounts for about 10% to 20% of all strokes and is associated with greater morbidity and mortality than ischemic strokes ^1^. Many modifiable risk factors, including prescription medications, have been identified for ICH. Cranial computed tomography (CT) is the imaging modality of choice for the diagnosis of acute ICH. Several CT parameters may predict hematoma expansion and neurologic worsening. These CT parameters, along with clinical criteria, have been used to create practical scoring schema to predict hematoma expansion. Despite high morbidity and mortality, there are no specific interventions that have been shown to improve clinical outcome after ICH. Over the last three years, important research has been carried out on the reversal of antiplatelet and oral anticoagulant (OAC)-associated ICH (OAC-ICH), acute blood pressure lowering, clearance of intraventricular hemorrhage (IVH) with intraventricular administration of alteplase, and decompressive craniectomy.

## Risk factors for intracerebral hemorrhage

**Figure 1.**
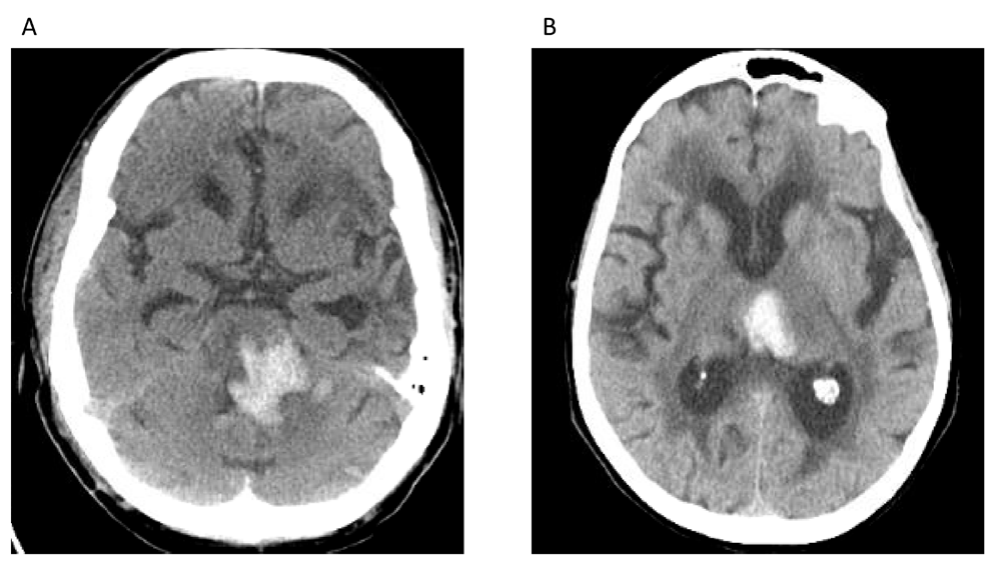
Intracerebral Hemorrhage due to arterial hypertension. ( **A**) Hyper-attenuating hemorrhage visualized in the pons. ( **B**) Hyper-attenuating hemorrhage centered in the left medial thalamus. Both locations are typical for hemorrhage related to long-standing hypertensive arteriopathy. These images constitute original, unpublished data obtained by the authors.

Modifiable risk factors for ICH include arterial hypertension ( Figure 1), excessive alcohol consumption, decreased low-density lipoprotein cholesterol, low serum triglyceride levels, prescription medications, current cigarette smoking, and drugs of abuse (for example, cocaine, heroin, amphetamines, and ephedrine) ^1^. Arterial hypertension is one of the most important modifiable risk factors as the crude prevalence among adults in the US has been estimated to be 45.6%; this is an increase from 31.9% based on prior definitions of arterial hypertension ^2^. Certain prescription medications, such as cyclooxygenase (COX) inhibitors, P2Y12 inhibitors, OACs, selective serotonin reuptake inhibitors (SSRIs), and statins, have also been associated with an increased risk of ICH.

Platelet inhibition related to COX-1 enzyme inhibitors (for example, aspirin) and P2Y12 purinoreceptor antagonists (for example, clopidogrel, ticagrelor, prasugrel, and cangrelor) is associated with increased ICH volume growth and worse clinical outcome ^3^. In a retrospective study of 82,576 patients, in-hospital mortality was higher among patients with ICH on dual antiplatelet therapy compared with no antiplatelet therapy ^3^.

A weak association between SSRIs/statins and ICH has been made. SSRIs cause antiplatelet effects by inhibiting serotonin reuptake into platelets ^4^. In a large retrospective cohort study, SSRI use was associated with an increased risk of ICH. This effect was most notable within the first 30 days of use and when used concomitantly with OACs ^4^.

The “Stroke Prevention by Aggressive Reduction in Cholesterol Levels” (SPARCL) trial suggested that statins potentially increase the risk for future ICH among patients with previous ischemic stroke or ICH ^5^. Since publication of the SPARCL study, data derived primarily from retrospective studies have not confirmed that statin use is a risk factor for ICH, and some have suggested that statin use is associated with improved mortality ^5^. In a meta-analysis of patients with prior ischemic stroke or ICH, there was a statistically significant risk reduction in poor functional outcome and all-cause mortality in both groups ^6^. Lobar localization of ICH, multiple bleeds, or multiple micro-hemorrhages may portend a high risk for statin use in patients with ICH. However, the posited association between statins/SSRIs and ICH requires more robust prospective evaluation.

ICH is the most feared complication of OAC use. The CROMIS-2 (“Cerebral microbleeds and intracranial hemorrhage risk in patients anticoagulated for atrial fibrillation after acute ischemic stroke or transient ischemic attack”) study was a recent prospective observational multicenter study of 1409 patients starting OAC following transient ischemic attack or ischemic stroke and identified risk factors for OAC-ICH ^7^. The presence of cerebral microbleeds and that of diabetes mellitus were the only two variables associated with ICH during the follow-up period. The authors noted that cerebral microbleeds may be a neuroimaging biomarker of a bleeding-prone arteriopathy. Interestingly, the commonly used “HAS-BLED” (hypertension, abnormal renal/liver function, stroke, bleeding history or predisposition, labile international normalized ratio [INR], elderly [age of at least 65 years], and drugs/alcohol concomitantly) score did not independently differentiate between patients with ICH and those without ICH. Precise identification of these risk factors and their magnitude of risk will promote patient-tailored management for secondary ischemic stroke prevention in the setting of atrial fibrillation. For example, isolation of the left atrial appendage may be an option for patients for whom the risk of ICH exceeds the risk of ischemic stroke ^8^. Cerebral amyloid angiopathy (CAA) is a non-modifiable risk factor for ICH and is associated with a higher risk of recurrent ICH than arterial hypertension–associated ICH. Apolipoprotein E (ApoE) alleles are important genetic risk factors for the development of sporadic CAA ^9^. For example, the number of ε4 alleles relates to clinical severity of CAA-related lobar ICH ^9^. The ε2 is also associated with an increased risk of CAA-related lobar ICH ^9^.

In patients with lobar ICH, pathologically graded moderate or severe CAA was independently associated with CT findings of subarachnoid hemorrhage and “finger-like” projections and an ApoE genotype. These diagnostic criteria may be helpful in determining CAA-associated ICH ^10^.

## Imaging predictors of hematoma enlargement

Hematoma enlargement is a known risk factor for poor outcome in patients with ICH ^11, 12^. Therefore, imaging predictors of hematoma expansion continue to be an important area of research. The computed tomography angiography (CTA) “spot sign” has been described as a predictor for hematoma expansion and poor functional outcome ^13^. One limitation of relying on CTA to predict hematoma expansion is the generalizability to medical centers where early CTA is not readily available. Importantly, the CTA “spot sign” is inversely related to ICH onset-to-CTA time ^14^. Additionally, in a large patient-level meta-analysis, the presence of a CTA spot sign did not significantly add to a prediction model of hematoma expansion based on established predictors ^15^.

Several recent studies have examined specific CT parameters to predict hematoma expansion. Li *et al*. described a CT finding called the “blend sign” to predict hematoma expansion ^16^. The “blend sign” was defined as blending of a hypo-attenuating area within the hyper-attenuated ICH with a well-defined margin. In their cohort, time to baseline CT scan, initial hematoma volume, and presence of “blend sign” on baseline CT were independent predictors of hematoma growth ^16^. The “blend sign” also has comparative positive predictive value to the spot sign for predicting neurologic deterioration, making it a useful CT marker when CTA is not available ^17^.

**Figure 2.**
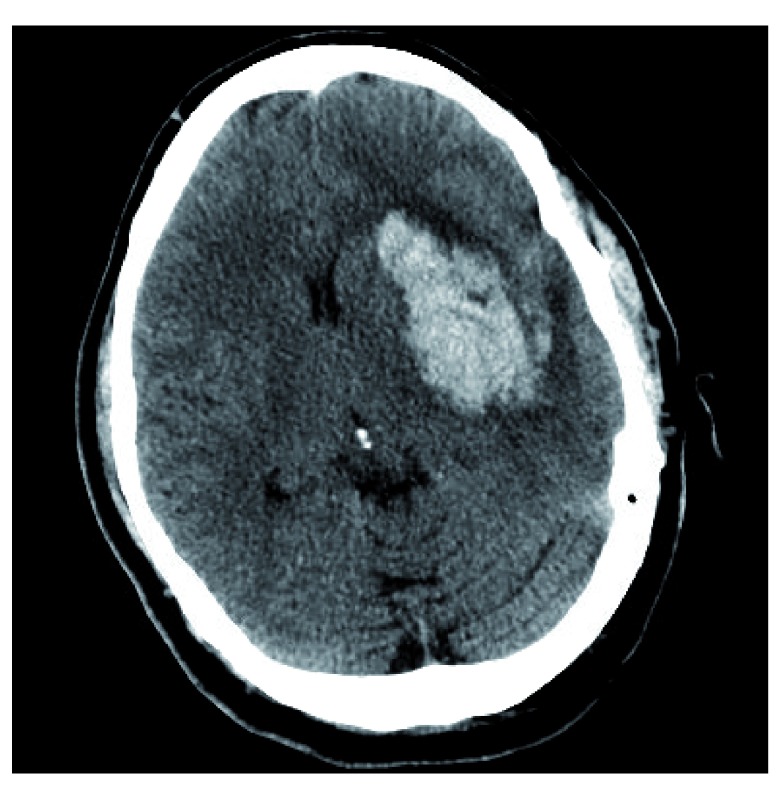
Left putaminal hemorrhage with positive “black hole sign”. A well-delineated margin is appreciated between the hypo-attenuating, round black hole and the hyper-attenuating hemorrhage. This image constitutes original, unpublished data obtained by the authors.

Analogous to the “blend sign”, the “black hole sign” is another CT sign to predict hematoma expansion. The “black hole sign” is defined as a hypo-attenuating area encapsulated within the hyper-attenuating ICH with a clearly defined border ( Figure 2). Similar to the “blend sign”, time to baseline CT scan, initial hematoma volume, and presence of a “black hole sign” on baseline CT were independent predictors of hematoma growth ^12^. Notably, both signs are reflections of heterogeneity within the ICH bed. The presence of these signs may represent bleeding at two distinct time periods ^5^. Additionally, Morotti *et al*. have shown that using a combination of the CTA spot sign and identification of any hypodensity within the hematoma on CT is superior to predicting hematoma expansion than either sign alone ^18^.

## Scores to predict hematoma expansion

Scoring schema that can accurately predict hematoma expansion are practical tools for future research. Interventions that do not show efficacy in an indiscriminate sample of patients with ICH may show efficacy in patients at high risk for hematoma expansion. Previous studies ^19, 20^ did not discriminate enrollment for high and low risk of ICH expansion; therefore, this may be an important source of residual confounding despite randomization. A practical baseline score may alleviate this source of confounding or allow post-hoc adjusted analysis. Three scoring systems that have been introduced to predict hematoma expansion are the “BRAIN” score ^21^ ( Table 1), the “BAT” score ^18^ ( Table 2), and a 9-point prediction score by Brouwers *et al*. ^22^ ( Table 3).

**Table 1.** “BRAIN” score.

Components	Points
Baseline intracerebral hemorrhage (ICH) volume	Milliliters per score: ≤10 = 0; 10–20 = 5; >20 = 7
Recurrent ICH	Yes = 4
Anticoagulation with warfarin at onset	Yes = 6
Intraventricular extension	No = 0; Yes = 2
Number of hours to baseline computed tomography	≤1 = 5; 1–2 = 4; 2–3 = 3; 3–4 = 2; 4–5 = 1; >5 = 0
Total score	0–24

**Table 2.** “BAT” score.

Components	Points
Blend sign	Yes = 1
Any hypodensity	Yes = 2
Time from onset to non-contrast computed tomography (<2.5 hours, ≥2.5 hours)	<2.5 hours = 2
Total score	0–5

**Table 3.** Nine-point prediction score for hematoma expansion by Brouwers *et al.* ^22^.

Components	Points
Warfarin use	Yes = 2
Time to initial computed tomography (CT)	≤6 hours = 2
Baseline intracerebral hemorrhage volume	<30 mL = 0; 30–60 mL = 1; >60 mL = 2
CT angiography spot sign	Absent = 0; present = 3; unavailable = 1
Total score	0–9

The “BRAIN” score is 24-point score based on baseline ICH volume, recurrent ICH, anticoagulation with warfarin at symptom onset, intraventricular extension, and number of hours to baseline CT from symptom onset. The maximum score, 24, retrospectively predicted hematoma expansion with an 85.8% probability in a pilot randomized controlled trial ^21^. The “BAT” score is composed of three components: presence of a blend sign, any hypodensity within the hyper-attenuating hematoma, and time from onset to non-contrast CT. A dichotomized score (of 3 or more than 3) predicted hematoma expansion with 50% sensitivity and 89% specificity ^18^. Brouwers *et al*. have proposed a 9-point score derived from a multivariate analysis based on variables from a large retrospective cohort ^22^. Components associated with hematoma expansion include warfarin use, time to initial CT, baseline ICH volume, and presence of the CTA spot sign. A dichotomized score showed strong association with hematoma expansion ^22^.

## Hemostatic therapies

Given the poor outcome associated with hematoma expansion, a considerable amount of research has been dedicated to pharmacological hemostatic therapies. Importantly, pharmacological therapies that reduce hematoma growth do not always translate into improved functional outcome or survival. For example, recombinant activated factor VII (rFVIIa) is one such agent ^23^.

Recently, tranexamic acid (TXA) has been assessed as a hemostatic therapy in a phase 3 randomized controlled trial in patients with spontaneous ICH. The premise of this study was based on favorable data for the use of TXA in patients with traumatic intracranial hemorrhage. There was a smaller mean increase in hematoma volume in the TXA group compared with the placebo group. Similar to rFVIIa, there was no improvement in functional status or mortality at 90 days ^24^.

Compared with patients with spontaneous ICH, those on antithrombotic therapy have a greater likelihood of secondary hematoma expansion and an increased risk of death or poor functional outcome. Reversal agents play a major role in the management of these patients ^25^.

Platelet transfusion compared with placebo after primary ICH in patients on antiplatelet agents was assessed in the PATCH (“Platelet transfusion versus standard care after acute stroke due to spontaneous cerebral hemorrhage associated with antiplatelet therapy”) phase 3 randomized controlled trial ^26^. The majority of patients enrolled in the study used COX inhibitors only. The odds of death or dependence at 3 months was higher in the platelet transfusion group compared with the standard care group. The authors hypothesized that collateral perfusion around the ICH may be impaired and that platelet transfusion may increase the risk of thrombosis and subsequent lesion expansion. Additionally, platelet transfusions have pro-inflammatory effects and may enhance vascular permeability associated with inflammation and platelet consumption ^26^. The results of this study may not be generalizable to patients taking P2Y12 inhibitors, as only 5 such patients were enrolled in the study.

Anticoagulant reversal is the mainstay of therapy for patients with OAC-ICH ^25, 27, 28^. In a large retrospective study composed of 16 registries, patients with vitamin K antagonist (VKA)-associated ICH (VKA-ICH) who did not undergo reversal had the highest crude case fatality rate compared with those who received reversal agents ^28^. VKAs deplete coagulation factors II, VII, IX, and X. Repletion of factors may be accomplished by using either fresh frozen plasma (FFP) or prothrombin complex concentrate (PCC). The INCH (“Fresh frozen plasma versus prothrombin complex concentrate in patients with intracranial hemorrhage related to vitamin K antagonists”) study compared the safety and efficacy of FFP versus PCC in patients with VKA-ICH ^26^. 9% of patients in the FFP group compared with 67% of patients in PCC arm achieved the primary endpoint of an INR of not more than 1.2 within 3 hours of treatment initiation. PCC was also superior for secondary imaging endpoints, including hematoma expansion. Thromboembolic events occurred in seven patients in the PCC arm compared with one patient in the FFP arm. Importantly, the study did not find any statistical difference between clinical endpoints, although these were considered secondary endpoints and this study was inadequately powered to detect differences in clinical endpoints. This study highlights the importance of future adequately powered studies to compare clinical outcomes of PCC and FFP.

**Figure 3.**
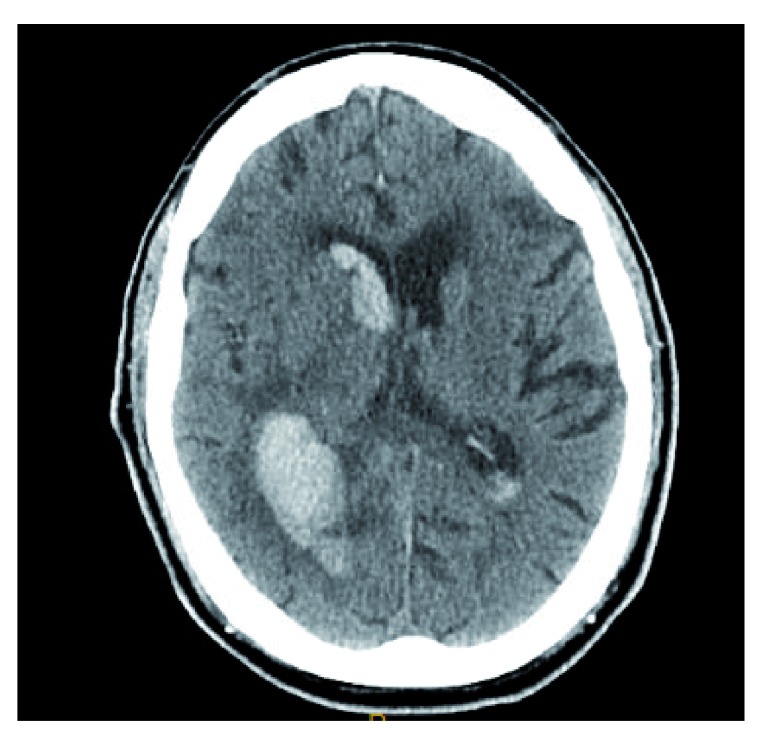
Rivaroxaban-related intracerebral hemorrhage. Hyper-attenuating hemorrhage is visualized in the left posterior temporal-parietal region with intraventricular extension. This image constitutes original, unpublished data obtained by the authors.

Direct oral anticoagulants (DOACs) are being increasingly used in patients with non-valvular atrial fibrillation (NVAF) because of an improved efficacy-to-safety ratio and fewer food and drug interactions compared with VKAs. Furthermore, DOACs have a substantially lower risk of ICH compared with VKAs; apixaban is associated with the lowest risk of ICH among the DOACs ^29^. In a prospective observational study, DOAC-ICH was analogous to VKA-ICH in regard to baseline hematoma volume and intraventricular extension ( Figure 3) ^30^. Three antidotes have been studied for the reversal of DOAC-ICH: idarucizumab, andexanet alfa, and ciraparantag.

A prospective cohort study evaluated the efficacy of idarucizumab, a monoclonal antibody fragment that binds dabigatran, in patients with dabigatran-related serious bleeding and those required to undergo an urgent procedure. This study enrolled 18 patients with ICH. This study showed a promising reversal effect. In the group of patients with serious bleeding, the dilute thrombin time normalized in 98% of patients ^31^. This study did not compare idarucizumab with alternative reversal agents and it remains unknown whether this reversal agent improves clinical outcomes. Interestingly, 34% of enrolled patients had a normal dilute thrombin time at the time of enrollment, highlighting the importance of well-designed randomized controlled trials to determine clinical effectiveness.

Andexanet alfa has also been assessed in patients with acute major bleeding ^32^. In total, 20 patients with ICH were enrolled in the efficacy population of the study. Following andexanet bolus, median anti-factor Xa activity decreased by 89% from baseline. However, median anti-factor Xa rose to 39% of baseline in the rivaroxaban group and 30% of baseline in the apixaban group 4 hours following an andexanet alfa infusion. Importantly, thrombotic events occurred in 18% of patients; stroke and deep venous thrombosis were the most common thrombotic events ^32^.

Ciraparantag is small synthetic water-soluble molecular entity that binds to heparin and oral direct factor Xa and factor II inhibitors by charge interaction and uncouples these drugs from their targets. In a phase 1 study of healthy subjects, a single intravenous dose of ciraparantag following 60 mg of edoxaban demonstrated full reversal of anticoagulation within 10 minutes; reversal was sustained for 24 hours ^33^.

## Acute blood pressure lowering

Blood pressure lowering is another potential target to prevent hematoma growth. Acute elevation in blood pressure after ICH is common and is a predictor of outcome ^34^. Hematoma expansion may be the means by which elevation of blood pressure portends early mortality and poor clinical outcome ^34^. The “Intensive Blood-Pressure Lowering in Patients with Acute Cerebral Hemorrhage” (ATACH-2) trial was a randomized controlled trial comparing intensive blood pressure lowering (defined as a systolic blood pressure goal of 110 to 139 mm Hg) with standard treatment (defined as a systolic blood pressure goal of 140 to 179 mm Hg) within 4.5 hours after symptom onset using intravenous nicardipine ^19^. Intensive blood pressure lowering did not result in a lower rate of death or disability compared with the standard treatment arm. Importantly, there was a statistically significant higher rate of renal adverse events in patients who were randomly assigned to the intensive treatment group. The ATACH-2 trial was consistent with an earlier randomized controlled trial by Anderson *et al*. which also did not find a reduction in death or disability in patients who underwent intensive blood pressure lowering ^20^. Furthermore, neither study showed a significant reduction in hematoma expansion—the presumed mechanism by which acute blood pressure lowering exerts its therapeutic action.

Subgroups from the ATACH-2 trial with imaging predictors of hematoma expansion have also been assessed. In a preplanned analysis of the ATACH-2 trial, patients who had a spot sign did not benefit from intensive blood pressure reduction similar to the entire study population ^35^. An additional post-hoc subgroup analysis of the same study found no benefit of intensive blood pressure lowering in patients with several CT markers of hematoma expansion ^36^.

In a prospective multicenter study of 600 patients with ICH and baseline magnetic resonance imaging, the authors found that changes in mean arterial pressure by 10 mm Hg were associated with diffusion-weighted imaging (DWI) lesions ^37^. These results are consistent with a prior study by Garg *et al*., who found an association between DWI lesions and greater acute blood pressure reductions ^38^. The authors note that patients with a greater burden of small-vessel disease may be deficient of normal cerebral autoregulation at the time of ICH and that acute blood pressure lowering may precipitate small-vessel ischemia ^37^. Importantly, there was an independent association between DWI lesions and 90-day outcomes ^37^.

The data on the efficacy of combined blood pressure lowering and anticoagulant reversal for patients with OAC-ICH remain sparse. A retrospective study of 1176 patients found that the combination of target INR reversal of less than 1.3 and systolic blood pressure lowering to less than 160 mm Hg was associated with lower rates of hematoma enlargement and in-hospital mortality ^11^. Although their data are biologically plausible, conclusions are limited by the confines of retrospective analysis without prospective confirmation. In summary, the optimal management of acute hypertension in patients with spontaneous non-traumatic ICH remains a therapeutic dilemma and an important area of future research.

## Intraventricular alteplase for intraventricular hemorrhage

In patients with spontaneous ICH, the presence of IVH portends a higher mortality compared with those without IVH. Disruption of IVH may relieve acute obstructive hydrocephalus and neurotoxicity associated with hemorrhage. The CLEAR III (“Clot Lysis: Evaluating Accelerated Resolution of Intraventricular Hemorrhage”) trial was a randomized controlled trial that assessed whether pharmacological disruption of IVH via intraventricular alteplase improves outcomes ^39^. The vast majority of patients represented in the trial were patients with thalamic hemorrhages with secondary intraventricular rupture. Patients who received alteplase had smaller IVH volumes and a shorter duration to ventricular patency. Despite these radiographic improvements, there was no improvement in 90-day functional outcomes in the patients who received intraventricular alteplase.

Staykov *et al*. have studied adjuvant lumbar drainage in patients with IVH treated with intraventricular alteplase ^40^. In an open-label, parallel-group study, patients were randomly assigned to either adjuvant lumbar drainage or no lumbar drainage after IVH resolution through intraventricular alteplase. There was a statistically significant reduction in shunt dependency in the lumbar drainage arm ^40^. This pilot study supports further prospective assessment of this intervention in a larger randomized controlled trial using patient-centered clinical endpoints.

## Surgical therapies for intracerebral hemorrhage

Two landmark randomized controlled trials did not show benefit of surgical intervention compared with medical management in patients with ICH. Patients with large ICH may develop significant delayed edema and this may cause secondary neurological injury due to mass effect. Decompressive craniectomy may mitigate the effects of delayed edema. In a retrospective study of 73 ICH patients who underwent decompressive craniectomy, 29% of patients had a favorable neurological outcome. This was noted to be superior to a historical cohort with lower ICH severity. Meaningful conclusions, however, are confined to retrospective analysis and support the need for further prospective data ^41^. The SWITCH (“Swiss Trial of Decompressive Craniectomy Versus Best Medical Treatment of Spontaneous Supratentorial Intracerebral Hemorrhage”) trial is an ongoing randomized controlled trial that will attempt to further define the role of decompressive hemicraniectomy in patients with supratentorial ICH.

There are ongoing studies of minimally invasive surgical techniques to alleviate surgical trauma that may offset the benefit of hematoma evacuation. The MISTIE (“Safety and efficacy of minimally invasive surgery plus alteplase in intracerebral hemorrhage evacuation”) study was a phase 2, open-label trial that evaluated catheter-based removal of ICH in patients with spontaneous, non-traumatic, supratentorial ICH ^42^. Catheters were placed under direct imaging guidance and clot aspiration was performed; alteplase then was administered through the catheters, allowed to dwell, and then re-opened for gravitational drainage. The minimally invasive surgery (MIS)-with-alteplase group had significantly smaller hemorrhage volumes compared with the medical management arms. Of note, for a composite safety endpoint of any cranial bleeding (symptomatic or asymptomatic) of more than 5 mL 72 hours after the last alteplase dose, there were significantly more events in the MIS-with-alteplase group. The landmark phase 3 study, recently published online, showed no benefit in the primary efficacy endpoint (mRS 0–3 at 365 days) ^43^. 

In a meta-analysis of 14 studies, MIS was significantly associated with reduced death or significant functional impairment compared with both medical treatment and conventional craniotomy. This meta-analysis included multiple types of MIS and had relatively low heterogeneity ^44^.

## Future directions and conclusions

ICH remains an important cause of morbidity and mortality. To date, no specific therapies directed toward ICH have been shown to improve functional outcome on the basis of randomized controlled trials. Despite this, patients who underwent maximal medical and surgical management have better outcomes than would be predicted on the basis of current prognostic scores ^45, 46^.

A lack of specific therapies may be reflective of either currently ineffective therapies or effective therapies that may only be beneficial in certain subgroups of patients with ICH. Therefore, further research into the identification of these subgroups may be an important step forward. For example, study groups composed of only patients with a high risk of hematoma expansion or exclusion of patients with a low risk of hematoma expansion may be beneficial. The use of clinical and radiographic parameters that have been established may allow for this.

Further emphasis on more translational research for acute ICH therapies may be warranted. For example, pioglitazone, deferoxamine, and stem cell replacement have been assessed in translational research settings and led to clinical safety studies ^47^. Perihematomal edema (PHE) is associated with short-term functional outcome and one such potential therapeutic target ^48^. Prior studies employing dexamethasone and therapeutic hypothermia to reduce PHE have revealed excess harm, and an ongoing study using deferoxamine, an iron chelator, has suspended enrollment because of an increased incidence of acute respiratory distress syndrome ^49^.

Complementary to research for acute ICH therapies, further research into risk factors for ICH may be necessary to lower the prevalence of this devastating stroke subtype. As prescriptions for COX-1 inhibitors, P2Y12 purinoreceptor antagonists, SSRIs, statins, VKAs, and DOACs are common, a more precise knowledge of the risk-to-benefit ratio of these medications and ICH is warranted for patient-tailored care. Additionally, optimal selection of patients for OAC for secondary stroke prevention will likely be further modified as further research is carried out on patients with high-risk features for ICH.
